# Zero-dose children and the immunisation cascade: Understanding immunisation pathways in low and middle-income countries

**DOI:** 10.1016/j.vaccine.2021.02.072

**Published:** 2021-07-22

**Authors:** Bianca O. Cata-Preta, Thiago M. Santos, Tewodaj Mengistu, Daniel R. Hogan, Aluisio J.D. Barros, Cesar G. Victora

**Affiliations:** a 1160 Marechal Deodoro, Pelotas, RS 96020-220, Brazil; b Chemin du Pommier 40, 1218 Grand-Saconnex, Geneva, Switzerland

**Keywords:** Immunisation, Vaccines, Child health, Healthcare disparities, Developing countries

## Abstract

**Introduction:**

Zero-dose prevalence refers to children who failed to receive any routine vaccination. Little is known about the “immunisation cascade” in low- and middle-income countries (LMICs), defined as how children move from zero dose to full immunisation.

**Methods:**

Using data from national surveys carried out in 92 LMICs since 2010 and focusing on the four basic vaccines delivered in infancy (BCG, polio, DPT and MCV), we describe zero-dose prevalence and the immunisation cascade in children aged 12 to 23 months. We also describe the most frequent combinations of vaccines (or co-coverage) among children who are partially immunized. Analyses are stratified by country income groups, household wealth quintiles derived from asset indices, sex of the child and area of residence. Results were pooled across countries using child populations as weights.

**Results:**

In the 92 countries, 7.7% were in the zero-dose group, and 3.3%, 3.4% and 14.6% received one, two or three vaccines, respectively; 70.9% received the four types and 59.9% of the total were fully immunised with all doses of the four vaccines. Three quarters (76.8%) of children who received the first vaccine received all four types. Among children with a single vaccine, polio was the most common in low- and lower-middle income countries, and BCG in upper-middle income countries. There were sharp inequalities according to household wealth, with zero-dose prevalence ranging from 12.5% in the poorest to 3.4% in the wealthiest quintile across all countries. The cascades were similar for boys and girls. In terms of dropout, 4% of children receiving BCG did not receive DPT1, 14% receiving DPT1 did not receive DPT3, and 9% receiving DPT3 did not progress to receive MCV.

Interpretation.

Focusing on zero-dose children is particularly important because those who are reached with the first vaccine are highly likely to also receive remaining vaccines.

## 1 Introduction

While there was a dramatic increase in coverage of new vaccines in low- and middle-income countries (LMICs) between 2000 and 2019, progress on extending routine immunisation services to ensure they reach all children has stagnated, and in many countries coverage levels remain below the goal of 90% established by the World Health Organization (WHO) [1], [2]. In the drive to leave no one behind with immunisation in the Sustainable Development Goals era, there is growing interest in reaching “zero-dose” children, a term that refers to children who have not received any routine vaccinations. In a review published in 2012 based upon surveys published up to 2007, it was estimated that 10% of all children living in LMICs had not received any immunisations [3]. This estimate, which to our knowledge has not been revisited in the intervening decade, was a stark finding given that immunisation is one of the most cost-effective measures for improving child survival globally [4].

The relative lack of progress in expanding routine immunisation services over the past decade – global coverage with the third dose of the vaccine against diphtheria, pertussis and tetanus (DPT) increased by one percentage point between 2010 and 2019 – suggests new efforts are needed to reach children and communities missing out on immunization, and, importantly, to ensure newly reached children benefit from the full complement of recommended vaccines [5]. To do this, it is important to update existing estimates of the frequency of zero-dose children in countries with recent data. Next, there is a need to understand how children move out of the zero-dose group towards full immunisation. Although there is ample information on coverage of individual vaccines, and on full immunisation coverage with all basic vaccines, to our knowledge there are no studies that describe what we refer to as the “immunisation cascade”. The cascade characterizes how, at population level, infants move from zero dose to full vaccination coverage by describing which vaccines are most likely to be received by children who have had a single vaccine, or combinations of two or more basic vaccines. This approach also provides more granular information on patterns of under-immunisation and dropout across key vaccination touchpoints in the first year of life. This analysis focused on four vaccines, namely Bacille Calmete-Guérin (BCG), vaccine against poliomyelitis (polio), DPT, and measles-containing vaccine (MCV).

Studies of coverage with different vaccines [6], of full immunisation coverage [7], and of zero dose [3] show wide inequalities both between and within countries, driven by the socioeconomic status of children’s families. Yet, there are no comprehensive multi-country analyses of inequalities in zero-dose children, nor on the progression from zero dose to full immunisation. We explored the magnitude of inequality according to the family’s socioeconomic position, the child’s sex, and place of residence in 92 countries with comparable national surveys carried out since 2010.

Our analyses are directly relevant to SDGs number 3 (to ensure healthy lives and promote well-being for all at all ages, including vaccination coverage and universal health coverage), 10 (to reduce inequalities) and 17 (to increase the availability of data disaggregated by income, sex and residence) [8] and to WHO 2030 Immunisation Agenda [9].

## 2 Methods

### 2.1 Data sources and study sample

We analysed nationally representative data from Demographic and Health Surveys (DHS) and Multiple Indicator Cluster Surveys (MICS) conducted in 92 LMICs between 2010 and 2019. MICS and DHS are highly comparable in terms of sampling, survey methods and questionnaires [10]. If two or more surveys were available for a country, we analysed the most recent one. Only countries with information on all vaccines under consideration were included in the analyses; for example, the 2018 survey from Suriname was excluded due to lack of information on BCG.

Information on immunisation status was extracted for children aged 12–23 months from two sources: vaccination cards or, if the child did not have a card or it was not available at the time of interview, the mother’s/caregiver’s report. We extracted data on the age group 15–26 months in one country (Moldova, 2012) where the measles-containing vaccine was given at 15 months, and on the age group 18–29 months in seven countries (Jamaica, 2011; Ukraine, 2012; Costa Rica, 2011; Tunisia, 2011; Bosnia and Herzegovina, 2011; Macedonia, 2011; and Egypt, 2014) where the vaccine was offered at 18 months. In Cuba, two polio vaccine doses are recommended during the first year of life, so that a child with two doses was treated as if it had received the third dose.

### 2.2 Outcomes

Four main outcomes were analysed, all based on the four basic vaccines: BCG, polio, DPT and measles containing vaccines (MCV). For polio, we considered both oral polio vaccine (OPV) and inactivated polio vaccine (IPV) and doses given at birth were not considered in the analyses [11]. For DPT, we considered any vaccine containing diphtheria, pertussis and tetanus antigens (e.g. pentavalent vaccine). The WHO currently recommends that the BCG vaccine should be given at birth, three doses of polio and DPT should be given at 6, 10 and 14 weeks, and the measles vaccine should be given at 9–12 months, although some countries adopt somewhat different schedules [12].

The first outcome was the prevalence of zero-dose children, that is, those who had not received any doses of the four vaccines. The second outcome was the immunisation cascade, generated as a score ranging from 0 to 4. Each type of vaccine accounts for one point in the cascade regardless of the number of doses. For example, a child was coded as “1″ if either one, two or three doses of DPT/polio vaccine had been received. For each cascade level, we estimated the percentage of children with all possible combinations of different vaccines within the corresponding cascade level.

The third outcome was vaccine co-coverage with two vaccines, assessed in two ways. First, we calculated the conditional probability of receiving a vaccine given that another vaccine *was received.* Second, we estimated the conditional probability of receiving a vaccine given that the other vaccine *was not received*.

The fourth and last outcome was full immunisation coverage (FIC), defined as the proportion of children who had received one dose of BCG, three doses of polio, three doses of DPT and one dose of MCV.

### 2.3 Stratification variables

Data are presented for all countries and for three country income groups (low-, lower-middle and upper-middle income countries, according to information gathered from the World Bank Open Data repository [13]).

Analyses are also presented for the poorest and wealthiest quintiles, within each country. The wealth index was derived through principal components analysis (PCA) based on household assets and then divided into quintiles [14]. The first quintile represents the households with the poorest 20% of the sample, and fifth quintile the wealthiest 20%.

In supplementary materials, we present the vaccine co-coverage and the immunisation cascade in the three country income groups stratified by poorest and wealthiest quintiles. Also, we present the immunisation cascade stratified by sex of the child and area of residence (rural and urban).

### 2.4 Statistical analyses

The analyses were carried out with Stata version 16 and R version 4.0.2 and accounted for the multi-stage survey design, including sampling weights. Pooled results were weighted by national populations of children aged 12 to 23 months in 2015, which was the median year for all available surveys [13].

We performed Fisher’s exact tests to compare the percentage of children in each immunisation cascade level between the poorest and wealthiest quintiles. We used the Cochran–Armitage test for trend to compare the percentages of children in each immunisation cascade level between the three country income groups. Additionally, we performed chi-squared tests to compare the percentage of zero dose children in each income group between girls and boys, rural and urban residence, and wealth quintiles.

In agreement with World Health Organization recommendations, we treated children with missing information on vaccination as not immunized [11]. We performed a sensitivity analysis excluding countries with a zero dose prevalence higher than or equal to 10% and with 5% or more of zero dose due to missing information in all four vaccines (BCG, polio, DPT and MCV) and then, we calculate the pooled zero dose prevalence.

### 2.5 Ethical clearance

All analyses were based on anonymised datasets that are publicly available. The institutions that administered the surveys at national level were responsible for ethical clearance.

## 3 Results

We analysed data from 45 DHS and 47 MICS countries (Table S1 supplementary material), representing 67% of all LMICs in the world. The countries included 90% of all low-income, 73% of lower-middle and 46% of upper-middle income countries. The children analysed were mostly from lower middle-income countries (66.5%) and lived in rural areas (64.3%); 51.2% of the children were boys. Information about the sample and the data sources of all countries included in the analyses can be found in Supplementary Tables 1 and 2.

The sensitivity analysis showed that excluding South Sudan, the Democratic Republic of Congo and Sudan our zero dose estimate falls from 7.7% (95%CI: 7.4–7.9) to 7.0 (CI95% 6.7–7.2).

Zero-dose prevalence ranged from 5.2% in upper-middle income countries to 11.1% in low-income countries, with a total pooled prevalence of 7.7% in all countries studied (Table 1). Boys and girls were equally likely to belong to the zero-dose category. Zero-dose children were more frequently found in rural than in urban areas and in poorer households.

**Table 1:** Percent of zero-dose children according to sex, residence, and wealth quintiles, by country income groups.

		Country income groups											
		Low			Lower-middle			Upper-middle			All LMICs		
		Prevalence	95%CI		Prevalence	95%CI		Prevalence	95%CI		Prevalence	95%CI	
***Sex***	*Boys*	10.8%	10.0%	11.6%	7.1%	6.7%	7.5%	5.3%	4.4%	6.3%	7.7%	7.3%	8.0%
	*Girls*	11.3%	10.4%	12.3%	6.9%	6.5%	7.3%	5.1%	4.3%	6.0%	7.7%	7.3%	8.0%
	p value	0.3156			0.465			0.770			0.954		
***Residence***	*Urban*	6.5%	5.6%	7.5%	5.1%	4.7%	5.6%	4.5%	3.7%	5.5%	5.2%	4.8%	5.6%
	*Rural*	12.6%	11.8%	13.5%	7.9%	7.6%	8.4%	6.4%	5.6%	7.2%	9.0%	8.7%	9.4%
	p value	<0.001			<0.001			0.003			<0.001		
***Wealth quintile***	*Poorest*	17.3%	15.7%	18.9%	11.8%	11.1%	12.5%	7.5%	6.0%	9.3%	12.5%	11.9%	13.1%
	*2nd*	12.3%	11.0%	13.8%	8.7%	8.1%	9.5%	5.7%	4.8%	6.8%	9.1%	8.6%	9.7%
	*3rd*	10.3%	9.2%	11.6%	5.9%	5.3%	6.5%	4.8%	3.5%	6.4%	6.7%	6.3%	7.2%
	*4th*	8.1%	6.9%	9.5%	4.1%	3.7%	4.6%	3.4%	2.4%	4.8%	5.0%	4.5%	5.4%
	*Wealthiest*	5.1%	4.2%	6.1%	2.9%	2.4%	3.4%	3.7%	2.3%	6.0%	3.4%	3.0%	3.9%
	p value*	<0.001			<0.001			<0.001			<0.001		
**Total**	*–*	11.1%	10.4%	11.8%	7.0%	6.7%	7.3%	5.2%	4.6%	5.9%	7.7%	7.4%	7.9%

* Chi-squared test for linear trend.

**Table 2:** Co-coverage with the four vaccines according to cascade level (all countries combined).

Cascade level*	Vaccines combinations	% of all children	n
0 vaccines	None	7.7%	16,960
1 vaccine	BCG	1.1%	2469
	Polio	1.9%	3216
	DPT	0.1%	251
	MCV	0.2%	378
2 vaccines	BCG + Polio	1.4%	2976
	BCG + DPT	0.6%	1396
	BCG + MCV	0.3%	638
	Polio + DPT	0.8%	1632
	Polio + MCV	0.4%	780
	DPT + MCV	0.1%	199
3 vaccines	BCG + Polio + DPT	10.7%	23,787
	BCG + Polio + MCV	1.0%	2497
	BCG + DPT + MCV	1.9%	4139
	Polio + DPT + MCV	1.0%	2232
4 vaccines	BCG + Polio + DPT + MCV	70.9%	147,591

* “vaccines” refers to having at least the first dose of a particular vaccine; MCV: measles containing vaccine; DPT: diphteria-pertussis-tetanus; BCG: Bacille Calmette-Guérin.

**Fig. 1:**
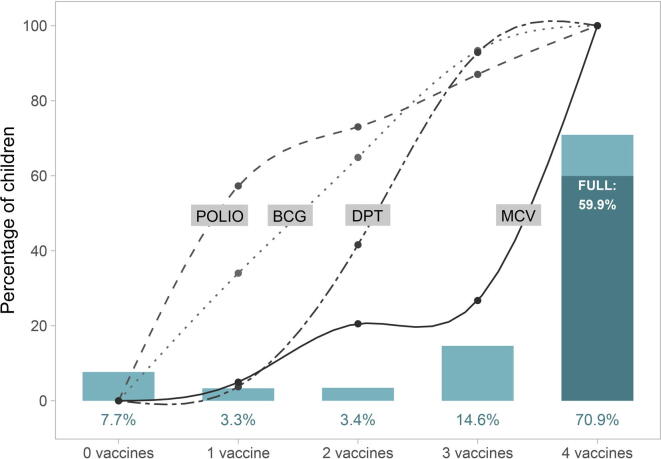
The immunisation cascade: vaccines delivered to children according to how many different types of vaccines each child received (all countries combined). Legend: FULL: full immunisation coverage; MCV: measles containing vaccine; DPT: diphteria-pertussis-tetanus; BCG: Bacille Calmette-Guérin.

**Fig. 2:**
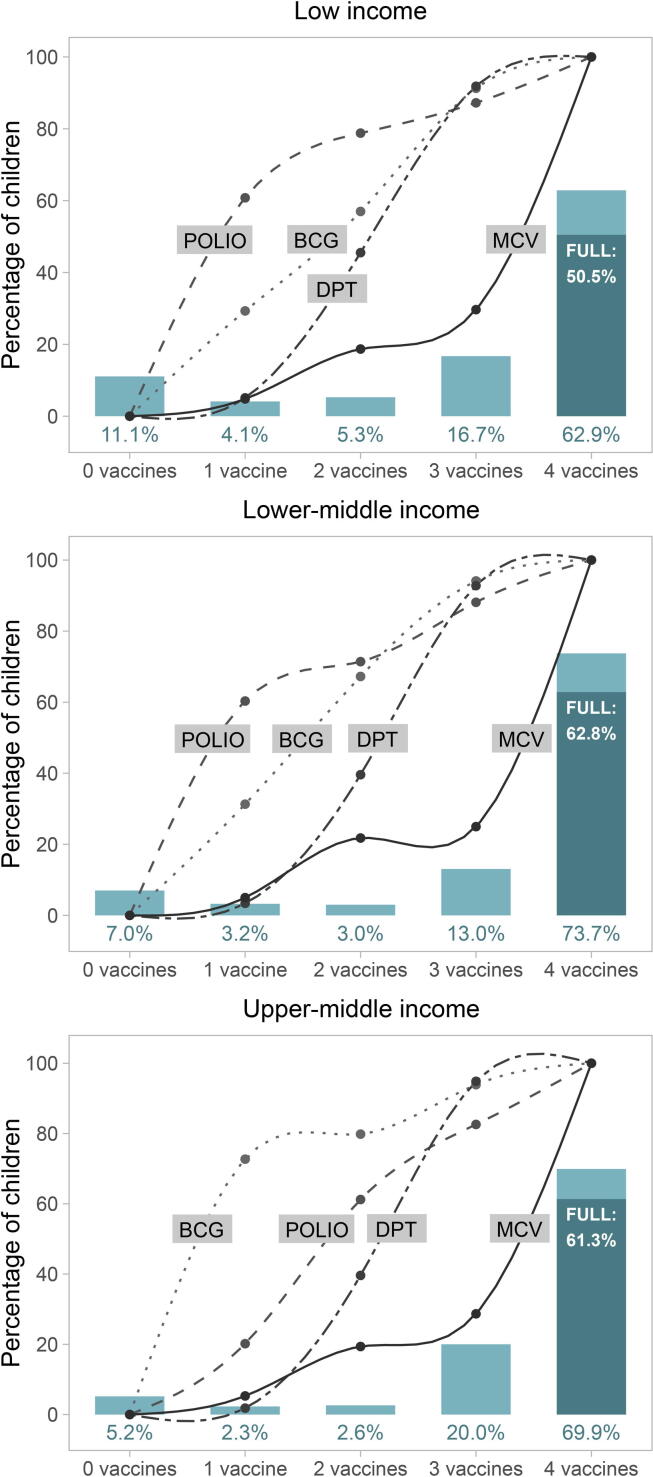
The immunisation cascade, according to country income groups. Legend: FULL: full immunisation coverage; MCV: measles containing vaccine; DPT: diphteria-pertussis-tetanus; BCG: Bacille Calmette-Guérin.

**Fig. 3:**
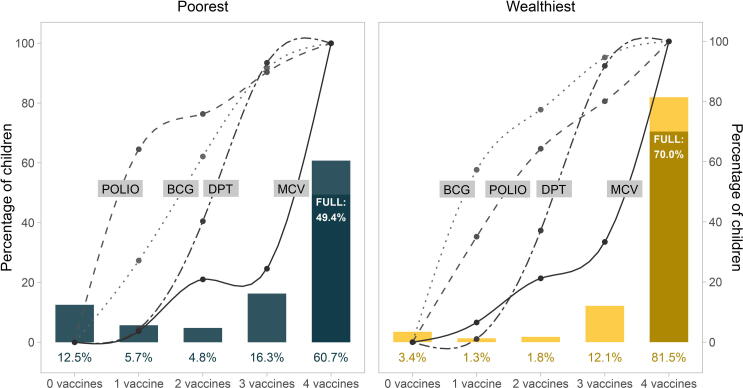
The immunisation cascade in the poorest and wealthiest quintiles (all countries combined). Legend: FULL: full immunisation coverage; MCV: measles containing vaccine; DPT: diphteria-pertussis-tetanus; BCG: Bacille Calmette-Guérin.

In the immunization cascade plots (Fig. 1, Fig. 2, Fig. 3), the bars represent the percentages of children in each cascade level (children who did not receive any doses of either of the 4 vaccines, children who received at least one dose of 1, 2,3, or all four types of vaccines). The darker portion of the last bar represents the percentage of children who have been fully immunised, that is, received all recommended doses of all four vaccines. The lines represent the percent of children in each level of the cascade who had received at least one dose of each of the four vaccines.

For all children studied, the five levels of the cascade are shown in Fig. 1. Being zero dose is more common than receiving at least one dose of exactly 1 or 2 vaccines; in other words, most children tend to be either zero dose or receive at least the first dose of 3 or more vaccines. Polio was the most frequent vaccine for children who received at least one dose of only one vaccine and MCV was the least frequent vaccine for children who had received at least one dose of one, two or three vaccines.

The last bar in Fig. 1 show that, for the 92 countries combined, 70.9% of all children received at least one dose of the four different vaccines. Full immunisation coverage, that is, all recommended doses of the four vaccines, was achieved by 59.9% of all children. The ratio between the two estimates suggests 15.5% of children who receive BCG, MCV and at least the first dose of polio and DPT vaccines do not progress to being fully vaccinated against polio and DPT. Of the 92.3% of children who received at least one vaccine, about three quarters (76.8%) received all four types, and two thirds (64.9%) were fully immunised.

All possible combinations of vaccines in each level of the cascade are shown in Table 2. BCG and polio were the most frequent combination for children with two different vaccines, while BCG, polio and DPT were the most common combination for those with three vaccines.

Table 3 shows the national coverage (bottom row) of each vaccine and of FIC, and the conditional probabilities of receiving each vaccine when another vaccine had been received (Table 3a), or when the other vaccine had not been received (Table 3b). For polio and DPT vaccines, the first and third dose are included in the table. Darker cells show higher probability levels. By definition, all children with FIC had a probability of 100% of having received all vaccines (Table 3a, first row). Children who receive MCV are very likely to have received BCG (98%), DPT1 (98%), and polio1 (97%), but only 84–86% of children receiving those vaccines also receive MCV. Over 80% of children who receive a third dose of DPT or polio are fully immunized, compared to only 68% of children who receive BCG. Children who do not receive DPT1 only have an 13% chance of receiving MCV.

**Table 3:** Co-coverage with the four basic vaccines during the first year of life.

FULL: full immunisation coverage; MCV: measles containing vaccine; DPT: diphteria-pertussis-tetanus; BCG: Bacille Calmette-Guérin.

Results from Table 3a may also be interpreted in terms of drop-out. Based on these surveys, 14% of children receiving the first dose of DPT do not receive the third dose of DPT in LMICs, and 19% of children receiving the first dose of polio do not receive the third dose of this vaccine. The survey data also allows for calculation of cross-vaccine measures of drop-out. For example, considering the five health system touchpoints across the first year of life for routine immunisation, DPT drop-out is greater than drop-out across vaccines, with 4% of children receiving BCG not receiving DPT1, 14% of those receiving DPT1 not receiving DPT3, and 9% of children receiving DPT3 not progressing to receive MCV. Drop-out patterns by wealth quintile are presented in Supplementary Table 3. Based on data pooled across all countries, the drop-out rates in the wealthiest quintile equal 3% for BCG to DPT1, 9% from DPT1 to DPT3, and 7% for DPT3 to MCV, compared to 6%, 19% and 12%, respectively in the poorest quintile.

Table 3b shows the probability of receiving a given vaccine (or vaccine doses, for polio and DPT) among children who did not receive another vaccine. BCG, Polio1 and DPT1 were the three vaccines most likely to be received by children who failed to receive the other vaccines.

Analyses of the immunisation cascade by country income groups reveals interesting patterns (Fig. 2). The most frequent vaccines for children with only one vaccine were polio in low and lower-middle, and BCG in upper-middle-income countries. MCV was the least frequent vaccine in all groups of countries, showing that children who receive MCV tend to have also received the three remaining vaccines. FIC was lowest in low-income countries (50.5%), but similar in lower- and upper-middle income countries, 62.8% and 61.3%, respectively (p value for trend < 0.0001).

Averaging results for all children in a group of countries masks important inequities within and across countries. Fig. 3 shows that, with all countries grouped together, children from the families in the poorest quintile presented zero-dose prevalence (12.5%) that was over three times higher that of children from families in the wealthiest quintile (3.4%, p value < 0.0001). For all cascade levels and for FIC, the poorest performed worse than the wealthiest children (p value < 0.0001). Polio was the first vaccine to reach poor children, and BCG the first to reach those from wealthy families.

Supplementary figure 1 shows similar analyses stratified by family wealth and according to groups of countries. Differences between rich and poor children in terms of zero dose and FIC are most marked in low and lower-middle income countries. Polio was the most frequent vaccine received by children in low-income countries and by poor children in lower-middle-income countries, while BCG was the most frequent vaccine for wealthy children in lower-middle-income and for all children in upper-middle-income countries.

Supplementary figures 2 and 3 show the cascade results by sex of the child and urban/rural residence. There were no consistent differences among boys and girls. FIC was more common in urban than in rural areas, whereas the opposite was observed for children with zero, one or two vaccines. Among children with one or two vaccines, BCG was the most frequent in urban, and polio in rural areas.

## 4 Discussion

Our global analyses of the immunisation cascade represent a novel way to understand the dynamics of vaccination activities at country level and for subgroups defined on the basis of characteristics of the children and their families and subnational levels.

The 2012 publication by Bosch-Capblanch and colleagues, based on surveys carried out up to 2007, showed that 9.9% of LMIC children aged 12–59 months belonged to the zero-dose category [3]. We found a prevalence of 7.7% among children aged 12–23 months in the 92 countries with surveys from 2010 to 2019, with a range from 5.2% in upper-middle income countries to 11.1% in low income countries. Within all country income groups, there were important social gradients; for example, in low-income countries 17.3% of children whose families belonged to the poorest quintile did not receive any vaccines, compared to 5.1% of those in the wealthiest quintile in the same group of countries. As far as we are aware, there are no published analyses of within-country socioeconomic inequalities in zero-dose.

Across LMICs, only 6.7% of all children were placed in the second or third step of the cascade, that is, had received only one or two vaccines. The cascade, thus, shows a J-shaped pattern, with about 8% of children in the first step (zero-dose), about 3% each in the second and third steps, 15% in the fourth and 71% in the fifth step with four vaccines. This reflects a degree of polarization among children who have little or no access to immunisations, and those who receive most if not all vaccines. This suggests that moving children out of the zero-dose state is a particularly critical point in ensuring the entire population is fully immunised. The J-shaped pattern was particularly evident among children from poor families in low- and lower-middle-income country groups. This pattern was not observed in upper-middle income countries, where the proportion of zero-dose children was low in both the poorest and wealthiest quintile of families.

In low-income countries and among poor children in lower-middle income countries, polio stands out as the most frequent vaccine for children in the second or third step of the cascade. This finding is likely due to polio vaccination campaigns and lack of access to BCG due to low coverage with institutional delivery [15].For all other groups BCG is the most frequent vaccine. Being administered at the time of birth usually in the context of institutional delivery, BCG is also the vaccine that is most frequently provided in upper-middle income countries and in children from wealthier families in lower-middle income countries.

Co-coverage between two interventions is present when a given individual receives both of these [16]. Our conditional analyses in Table 3 shows a high frequency of co-coverage, that is, children who received any given vaccine are more likely to also receive other vaccines. BCG, DPT1 and polio1 stand out as the vaccines (or vaccine doses) that were most strongly associated with all vaccines. MCV, DPT3 and polio3, in contrast, showed weaker associations with the others.

This analysis highlights the importance of addressing drop-out across vaccination touchpoints in the first year of life to ensure zero-dose children, once reached, continue to become fully immunised. Children who receive BCG are quite likely to continue to receive DPT1, with a drop-out rate of 4%. However, drop out between DPT1 to MCV is 16% on average. There are large inequalities in drop-out rates, with drop-out being twice as high for children from poorest households, with a DPT1 to MCV drop-out rate of 18% compared to 9% in wealthier households.

Our results on socioeconomic inequalities regarding zero-dose prevalence and the immunisation cascade are well in line with the existing literature on inequalities in coverage, either with a specific vaccine or with full immunization [3], [6], [7]. Our findings (Supplementary Materials) of similar coverage patterns among boys and girls are consistent with an earlier analysis of 67 LMICs showing that the median difference was only 0.5 percent point, favouring boys, in coverage of several vaccines [6]. In terms of place of residence, this same study found that although urban coverage tended to be higher than rural coverage, differences were small except for a handful of countries [6]. In our pooled analyses, zero-dose prevalence was 9.0% in rural and 5.2% in urban areas. In spite of the presence of inequalities according to socioeconomic position and place of residence, a comparison of several indicators of coverage with reproductive, maternal, newborn and child health interventions showed that immunisation tends to be more equitable than interventions that require access to fixed health facilities [17].

The strengths of our analyses include the use of data from 92 countries, and novel ways of examining the immunisation cascade and co-coverage. Some limitations must be acknowledged. First, our analyses covered the majority of low- and lower-middle income countries, but only half of upper-middle income countries, as in these settings, surveys such as DHS or MICS are seldom conducted. Second, although we used the most recent survey from each country, the period covered ranges from 2010 to present, with a median year of 2015, therefore our results may not represent the current situation on vaccination in a given country. The analysis also does not account for possible declines in immunisation rates due to COVID-19 related disruptions. Per early assessments, disruptions to routine immunisation have been widespread and could result in large declines in immunisation coverage as well as further exacerbate inequalities [18]. Thirdly, although doses received during a campaign were computed, it was not possible to separate these from doses received during routine use of health services. Thus, it was not possible to investigate, for example, whether the polio doses reported for children with fewer vaccines were administered as part of campaigns. In agreement with standard international practice [11], information on immunisation was based on mother’s recall when a vaccination card was not available, a potential source of recall bias. Also, in agreement with recommendations, we treated children with missing information on vaccination as not immunized.

Regularly reaching zero-dose children and missed communities with routine immunisation, and ensuring those children become fully vaccinated, is a key goal of Immunisation Agenda 2030 and the Gavi Alliance’s 2021–2025 Strategy. This analysis suggests that reaching children with a first vaccine is important as most children who have been reached tend to receive several vaccines. The analysis also highlights that reaching zero-dose children and addressing barriers to under-immunisation will likely improve equity in immunisation coverage, as poorer children are much more likely to be zero-dose and have higher drop-out rates. Overcoming these challenges would help ensure no one is left behind with immunisation in the SDG era.

All authors attest they meet the ICMJE criteria for authorship.

## CRediT authorship contribution statement

**Bianca O. Cata-Preta:** Conceptualization, Software, Formal analysis, Writing - original draft, Writing - review & editing. **Thiago M. Santos:** Conceptualization, Software, Formal analysis, Visualization, Writing - review & editing. **Tewodaj Mengistu:** Conceptualization, Writing - review & editing. **Daniel R. Hogan:** Conceptualization, Writing - review & editing. **Aluisio J.D. Barros:** Conceptualization, Writing - review & editing, Supervision, Project administration. **Cesar G. Victora:** Conceptualization, Writing - original draft, Writing - review & editing, Supervision, Project administration.

## Declaration of Competing Interest

The authors declare that they have no known competing financial interests or personal relationships that could have appeared to influence the work reported in this paper.
