# Supplementary material for: Zero-dose children and the immunisation cascade: Understanding immunisation pathways in low and middle-income countries
Source: Vaccine. 2021 Jul 22;39(32):4564–70. doi: 10.1016/j.vaccine.2021.02.072 (PMC8314014; doi:10.1016/j.vaccine.2021.02.072)

Supplementary Table 1 – Data sources for the 92 countries included in the analyses.

| **ISO** | **Country** | **Year** | **Source** | **Income group** |
| --- | --- | --- | --- | --- |
| AFG | Afghanistan | 2015 | DHS | Low income |
| DZA | Algeria | 2012 | MICS | Upper middle income |
| AGO | Angola | 2015 | DHS | Upper middle income |
| ARM | Armenia | 2015 | DHS | Lower middle income |
| BGD | Bangladesh | 2014 | DHS | Lower middle income |
| BLZ | Belize | 2015 | MICS | Upper middle income |
| BEN | Benin | 2017 | DHS | Low income |
| BIH | Bosnia and Herzegovina | 2011 | MICS | Upper middle income |
| BFA | Burkina Faso | 2010 | DHS | Low income |
| BDI | Burundi | 2016 | DHS | Low income |
| CAF | CAR | 2010 | MICS | Low income |
| KHM | Cambodia | 2014 | DHS | Lower middle income |
| CMR | Cameroon | 2014 | MICS | Lower middle income |
| TCD | Chad | 2014 | DHS | Low income |
| COL | Colombia | 2010 | DHS | Upper middle income |
| COM | Comoros | 2012 | DHS | Low income |
| COD | Congo Democratic Republic | 2017 | MICS | Low income |
| COG | Congo Brazzaville | 2014 | MICS | Lower middle income |
| CRI | Costa Rica | 2011 | MICS | Upper middle income |
| CIV | Cote d’Ivoire | 2016 | MICS | Lower middle income |
| CUB | Cuba | 2014 | MICS | Upper middle income |
| DOM | Dominican Republic | 2014 | MICS | Upper middle income |
| EGY | Egypt | 2014 | DHS | Lower middle income |
| SLV | El Salvador | 2014 | MICS | Lower middle income |
| SWZ | Eswatini | 2014 | MICS | Lower middle income |
| ETH | Ethiopia | 2016 | DHS | Low income |
| GAB | Gabon | 2012 | DHS | Upper middle income |
| GMB | Gambia | 2018 | MICS | Low income |
| GHA | Ghana | 2017 | MICS | Lower middle income |
| GTM | Guatemala | 2014 | DHS | Lower middle income |
| GIN | Guinea | 2018 | DHS | Low income |
| GNB | Guinea Bissau | 2014 | MICS | Low income |
| GUY | Guyana | 2014 | MICS | Upper middle income |
| HTI | Haiti | 2016 | DHS | Low income |
| HND | Honduras | 2011 | DHS | Lower middle income |
| IND | India | 2015 | DHS | Lower middle income |
| IDN | Indonesia | 2017 | DHS | Lower middle income |
| IRQ | Iraq | 2018 | MICS | Upper middle income |
| JAM | Jamaica | 2011 | MICS | Upper middle income |
| JOR | Jordan | 2017 | DHS | Upper middle income |
| KAZ | Kazakhstan | 2015 | MICS | Upper middle income |
| KEN | Kenya | 2014 | DHS | Lower middle income |
| KIR | Kiribati | 2018 | MICS | Lower middle income |
| XKX | Kosovo | 2013 | MICS | Lower middle income |
| KGZ | Kyrgyzstan | 2018 | MICS | Lower middle income |
| LAO | Laos | 2017 | MICS | Lower middle income |
| LSO | Lesotho | 2018 | MICS | Lower middle income |
| LBR | Liberia | 2013 | DHS | Low income |
| MDG | Madagascar | 2018 | MICS | Low income |
| MWI | Malawi | 2015 | DHS | Low income |
| MDV | Maldives | 2016 | DHS | Upper middle income |
| MLI | Mali | 2018 | DHS | Low income |
| MRT | Mauritania | 2015 | MICS | Lower middle income |
| MEX | Mexico | 2015 | MICS | Upper middle income |
| MDA | Moldova | 2012 | MICS | Lower middle income |
| MNG | Mongolia | 2018 | MICS | Lower middle income |
| MNE | Montenegro | 2013 | MICS | Upper middle income |
| MOZ | Mozambique | 2015 | DHS | Low income |
| MMR | Myanmar | 2015 | DHS | Lower middle income |
| NAM | Namibia | 2013 | DHS | Upper middle income |
| NPL | Nepal | 2016 | DHS | Low income |
| NER | Niger | 2012 | DHS | Low income |
| NGA | Nigeria | 2018 | DHS | Lower middle income |
| MKD | North Macedonia | 2011 | MICS | Upper middle income |
| PAK | Pakistan | 2017 | DHS | Lower middle income |
| PAN | Panama | 2013 | MICS | Upper middle income |
| PNG | Papua New Guinea | 2016 | DHS | Lower middle income |
| PRY | Paraguay | 2016 | MICS | Upper middle income |
| PER | Peru | 2018 | DHS | Upper middle income |
| PHL | Philippines | 2017 | DHS | Lower middle income |
| RWA | Rwanda | 2014 | DHS | Low income |
| STP | Sao Tome and Principe | 2014 | MICS | Lower middle income |
| SEN | Senegal | 2017 | DHS | Low income |
| SRB | Serbia | 2014 | MICS | Upper middle income |
| SLE | Sierra Leone | 2017 | MICS | Low income |
| ZAF | South Africa | 2016 | DHS | Upper middle income |
| SSD | South Sudan | 2010 | MICS | Low income |
| PSE | State of Palestine | 2014 | MICS | Lower middle income |
| SDN | Sudan | 2014 | MICS | Lower middle income |
| TJK | Tajikistan | 2017 | DHS | Lower middle income |
| TZA | Tanzania | 2015 | DHS | Low income |
| THA | Thailand | 2015 | MICS | Upper middle income |
| TLS | Timor Leste | 2016 | DHS | Lower middle income |
| TGO | Togo | 2017 | MICS | Low income |
| TUN | Tunisia | 2018 | MICS | Lower middle income |
| TKM | Turkmenistan | 2015 | MICS | Upper middle income |
| UGA | Uganda | 2016 | DHS | Low income |
| UKR | Ukraine | 2012 | MICS | Lower middle income |
| VNM | Vietnam | 2013 | MICS | Lower middle income |
| YEM | Yemen | 2013 | DHS | Lower middle income |
| ZMB | Zambia | 2018 | DHS | Lower middle income |
| ZWE | Zimbabwe | 2019 | MICS | Low income |

Supplementary Table 2 – Sample characteristics.

|  |  | **n** | **Prevalence** |
| --- | --- | --- | --- |
| Sex | Male | 108034 | 51.2% |
|  | Female | 103107 | 48.8% |
| Age group | 12-14 months | 54495 | 25.8% |
|  | 15-17 months | 52578 | 24.9% |
|  | 18-20 months | 52381 | 24.5% |
|  | 21-23 months | 48784 | 23.0% |
|  | 24-29 months | 2903 | 1.8% |
| Area of residence | Urban | 73974 | 35.7% |
|  | Rural | 137167 | 64.3% |
| Wealth quintiles | Poorest | 54523 | 22.9% |
|  | Second | 47117 | 21.3% |
|  | Third | 42049 | 20.5% |
|  | Fourth | 36277 | 19.0% |
|  | Richest | 30014 | 16.4% |
| Income group | Low income | 60145 | 22.1% |
|  | Lower-middle income | 111924 | 66.5% |
|  | Upper-middle income | 39072 | 11.4% |

Supplementary Table 3. Co-coverage with the four basic vaccines during the first year of life in the poorest and wealthiest quintiles (all countries combined).


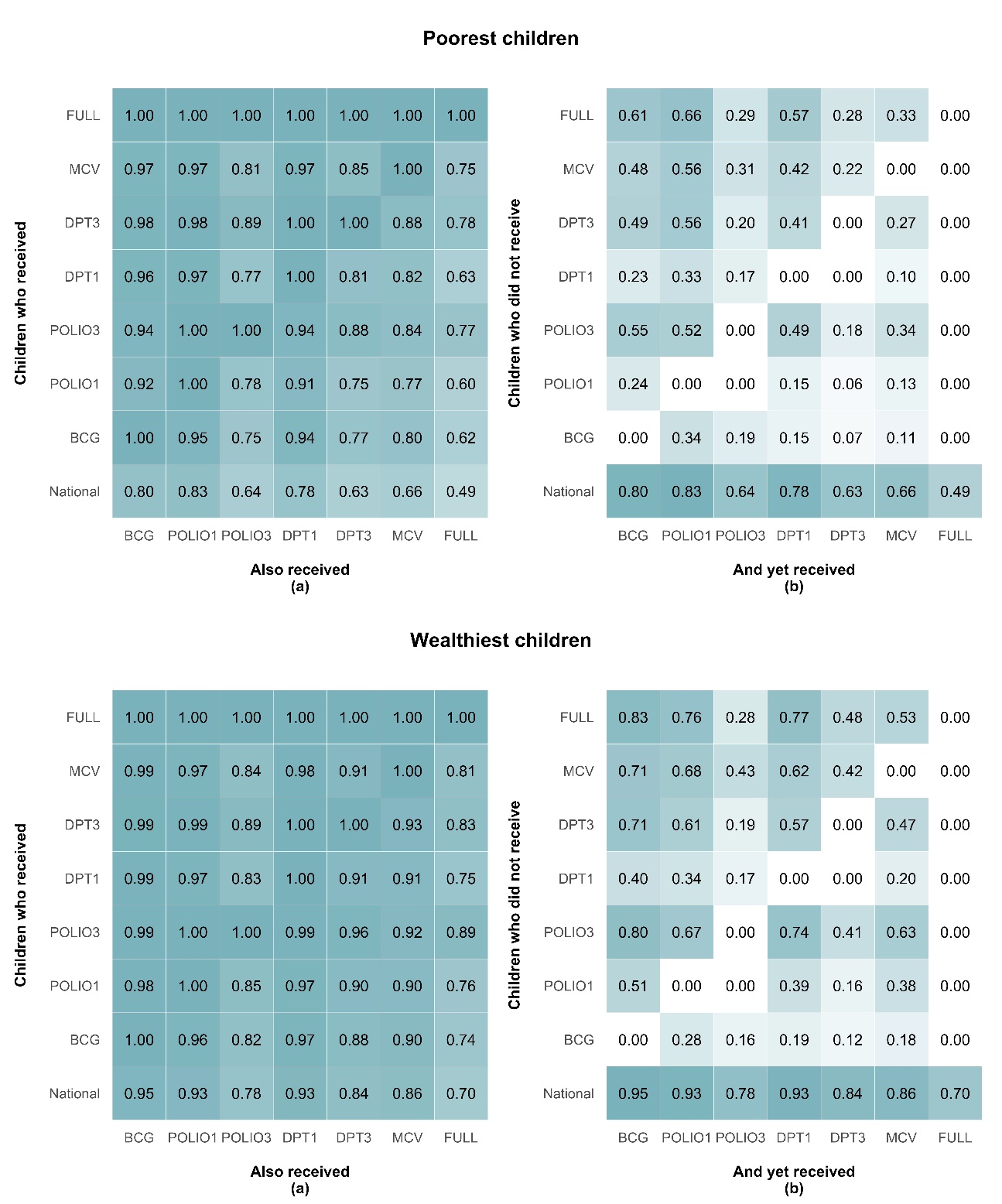


FULL: full immunization coverage; MCV: measles containing vaccine; DPT: diphteria-pertussis-tetanus; BCG: Bacille Calmete-Guérin.

Supplementary Figure 1 – The immunization cascade in the poorest and wealthiest quintiles by country income groups (all countries combined).


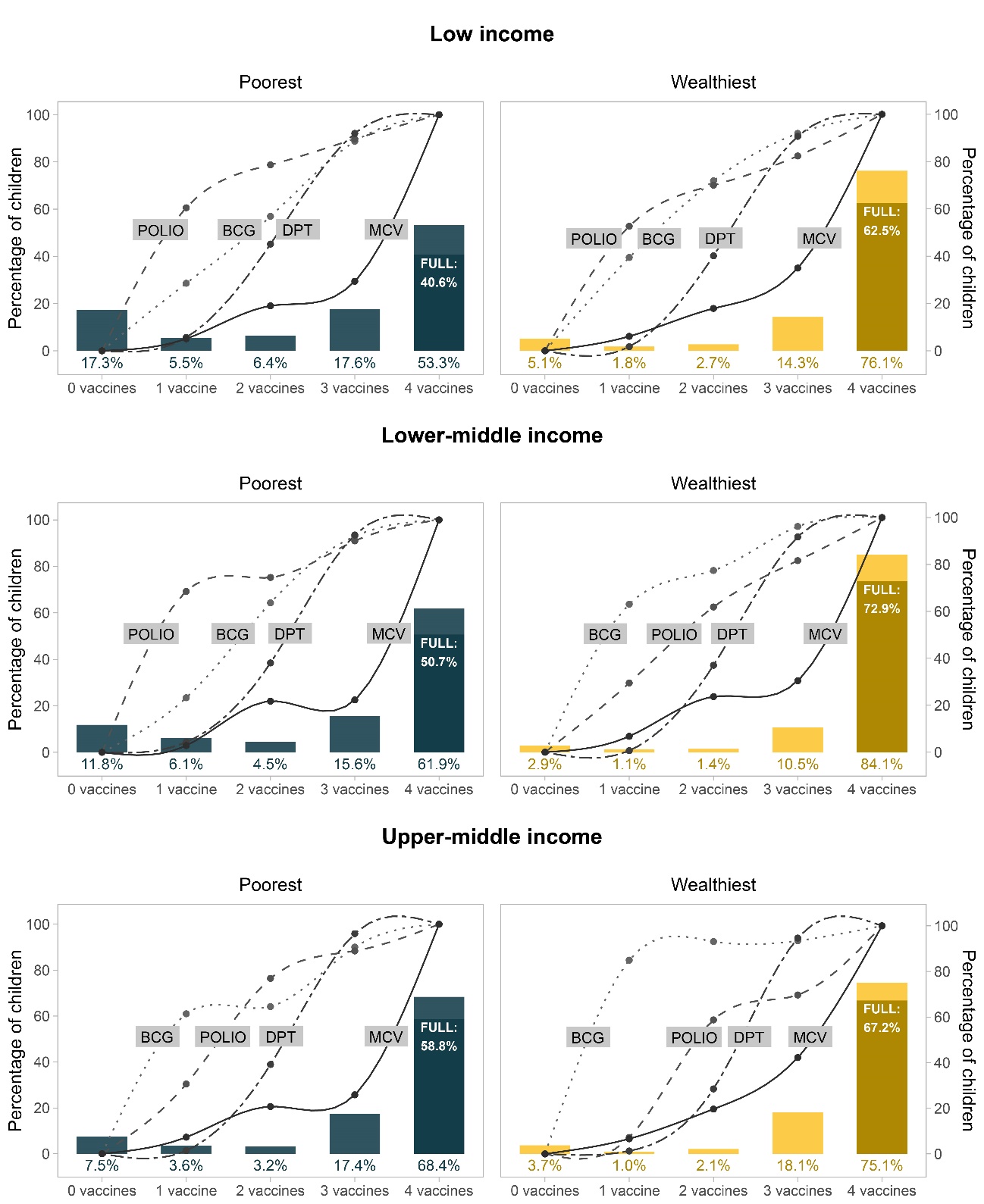


FULL: full immunization coverage; MCV: measles containing vaccine; DPT: diphteria-pertussis-tetanus; BCG: Bacille Calmete-Guérin.

Supplementary Figure 2 – The immunization cascade by sex of the child (all countries combined).


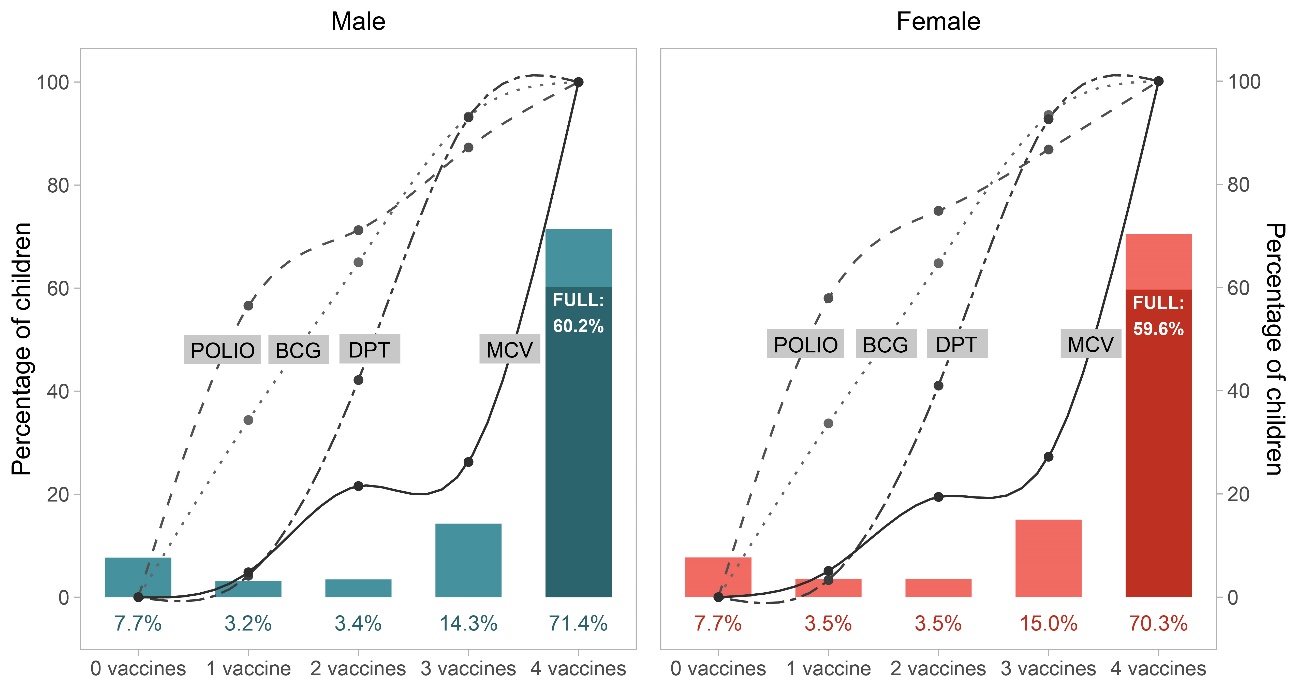


Supplementary Figure 3 – The immunization cascade by area of residence (all countries combined).


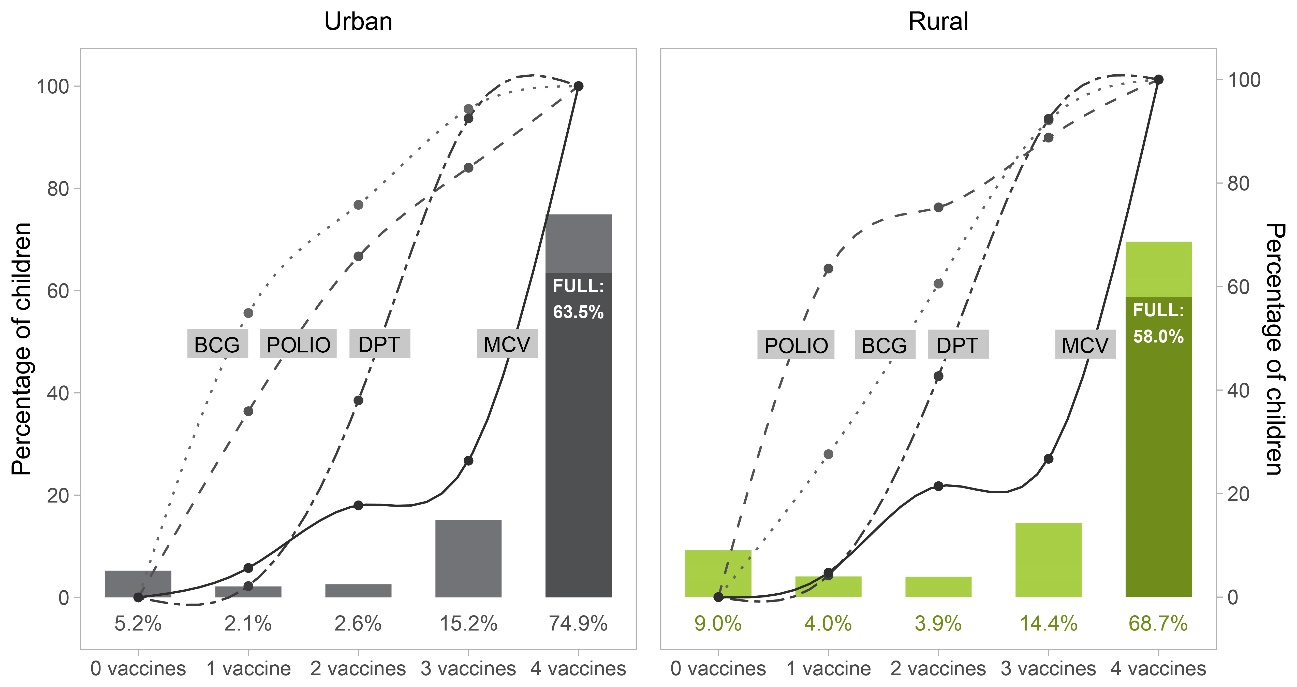

Supplement: Supplementary data 1 [file mmc1.docx]
